# Colonoscopy Leads to A Diagnosis of A Jejunal Gastrointestinal Stromal Tumour (GIST)

**DOI:** 10.4021/gr380w

**Published:** 2011-11-20

**Authors:** Justina J. Sam, Robert Mustard, Gabor Kandel, Geoffrey Gardiner, Hasan Ghaffar, Anish Kirpalani, Gary May, Young-In Kim

**Affiliations:** a Division of Gastroenterology, Department of Medicine, St. Michael’s Hospital and University of Toronto, Toronto, Ontario, Canada; b Division General Surgery, Department of Surgery, St. Michael’s Hospital and University of Toronto, Toronto, Ontario, Canada; c Department of Laboratory Medicine and Pathobiology, St. Michael’s Hospital and University of Toronto, Toronto, Ontario, Canada; d Department of Medical Imaging, St. Michael’s Hospital and University of Toronto, Toronto, Ontario, Canada

**Keywords:** Gastrointestinal stromal tumors, Intussusception, Gastrointestinal hemorrhage, Gastrointestinal neoplasms

## Abstract

Gastrointestinal stromal tumors (GISTs) are the most common mesenchymal tumors in the gastrointestinal (GI) tract, but are the least common of small intestinal malignant neoplasms. While GI bleeding is the most common clinical presentation of GISTs, intussusception and obstruction are uncommon, as GISTs rarely grow into the lumen. We describe an unusual case of a 50-year-old male who presented with intermittent obscure, overt GI bleeding requiring multiple hospital admissions and blood transfusions. His work-up included abdominal CT imaging, small bowel follow-through, gastroscopies, push enteroscopy, colonoscopies, and anterograde and retrograde double-balloon enteroscopies. Complicating his presentation were colonic angiodysplasias and the development of recurrent venous thromboembolism requiring anticoagulation. Within an hour after an apparently uncomplicated colonoscopy, he developed an acute abdomen secondary to a jejunal intussusception, which led to a laparoscopic small bowel resection and the diagnosis of a jejunal GIST. Given his GIST had no high-risk features, ongoing surveillance with abdominal CT imaging was arranged. This case illustrates the complex presentation and diagnostic difficulty of a jejunal GIST causing obscure, overt GI bleeding and this is the first reported case of a jejunal intussusception following colonoscopy. Due to its submucosal location, multiple endoscopic approaches had failed to diagnose the GIST prior to surgery.

## Introduction

Gastrointestinal stromal tumors (GISTs) are the most common mesenchymal tumors in the gastrointestinal (GI) tract, but account for only 1% of all GI malignancies [1]. GI bleeding is the most common clinical presentation of GISTs, as small GISTs can form solid subserosal or intramural masses, which can ulcerate or erode vessels. Intussusception and obstruction are uncommon, as GISTs rarely grow into the lumen [2, 3]. We describe a case of a patient with a jejunal GIST that presented with intermittent obscure, overt GI bleeding and jejunal intussusception, which presented shortly after an apparently uncomplicated colonoscopy. While there have been a few case reports describing colo-colonic intussusceptions following colonoscopy, this is the first reported case of small bowel intussusception after colonoscopy.

## Case Report

A 50-year-old male presented with a four-month history of intermittent melena and mild cramping abdominal pain. His past medical history included H. pylori eradication, renal calculi and a remote appendectomy. Initial investigations completed at a community hospital included a normal gastroscopy, a colonoscopy, which showed a small rectal polyp and internal hemorrhoids, and a negative small bowel follow-through. During the third month after the onset of his symptoms, he was readmitted to the same hospital for several episodes of bright red blood per rectum along with hypotension and presyncope and a hemoglobin of 69 g/L. Abdominal x-ray showed a non-specific bowel gas pattern. During this admission, he developed left leg pain and was found to have thrombosis of the left distal superficial femoral and popliteal veins and was started on dalteparin. A hypercoagulable work-up revealed that he was heterozygous for factor V Leiden. He was transfused a total of 4 units of packed red blood cells during his hospitalization, and his hemoglobin on discharge 14 days later was 81 g/L.

Within two days of his discharge from the community hospital, he developed recurrent melena with upper abdominal cramping and was admitted to our hospital with a hemoglobin of 79 g/L, which decreased to a nadir of 70 g/L the following day. He was transfused 1 unit of packed red blood cells resulting in a hemoglobin of 94 g/L. Push enteroscopy to the proximal jejunum demonstrated a small pre-pyloric erosion. CT abdomen was showed nodular mucosal thickening involving the cecum and ascending colon, with associated subtle stranding of the pericolonic fat with tiny lymph nodes. However, repeat colonoscopy was normal except for an 8 mm cecal nodule, which on biopsy showed mild cecitis. Anterograde double balloon enteroscopy showed focal granular changes in the jejunum, which corresponded on biopsy to patchy chronic inflammation within the lamina propria with no evidence of dysplasia. A subsequent retrograde double balloon enteroscopy was normal to the tattoo in the mid-ileum. While in hospital, anticoagulation was initially held, but restarted after the development of a right axillary vein thrombosis in the setting of a peripheral intravenous catheter. He was discharged home on warfarin with a hemoglobin of 76 g/L.

While at home, his melena recurred, leading to re-admission to our hospital within 4 weeks after his previous discharge. His hemoglobin on admission was 115 g/L. Repeat colonoscopy showed an actively oozing angiodysplasia in the ascending colon, treated with argon plasma coagulation, followed by an endoclip application, resulting in complete cessation of bleeding. Two additional angiodysplasias in the transverse colon and the rectum were treated with argon plasma coagulation. A 5 mm rectal polyp was removed with a cold snare and turned out to be a tubular adenoma. The terminal ileum was normal up to 20 cm from the ileocecal valve.

**Figure 1:**
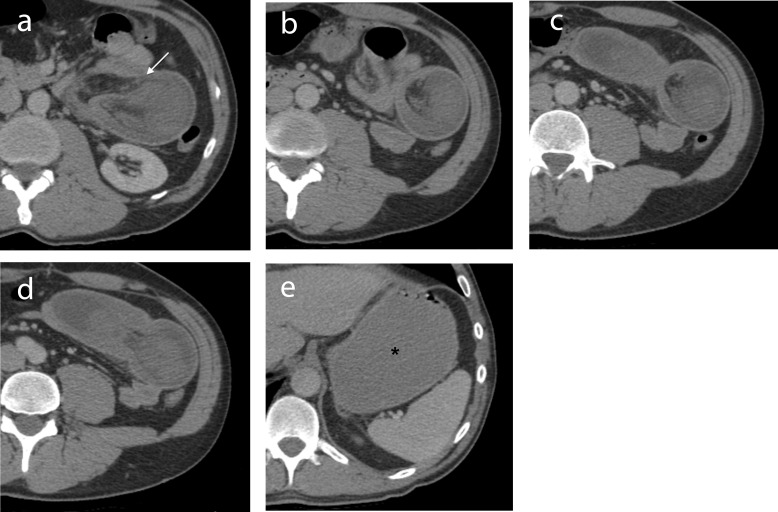
(a-d) IV contrast-enhanced axial CT images through the abdomen demonstrate a jejunal intussusception on the left side with proximal obstruction. The dilated intussuscepiens contains the intussusceptum along with mesenteric fat and vessels (arrow). (e) The stomach is distended (^*^). The 2.5 cm GIST causing the intussusception is not seen on CT.

**Figure 2:**
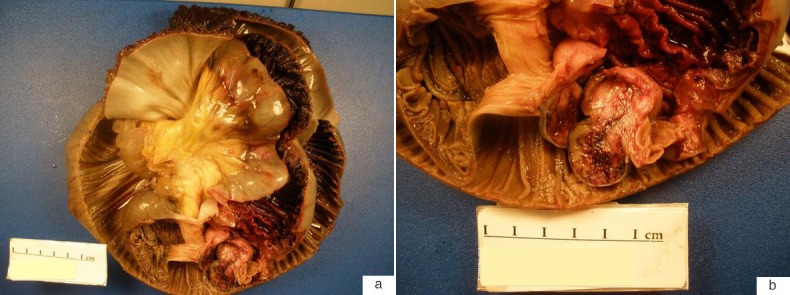
(a) The gross specimen consisted of an intussuscepted segment of small bowel. (b) A 2.5 cm solitary, well-circumscribed neoplasm with hemorrhagic cut surfaces was present at the base of the intussusception arising from within the submucosa and muscularis propria. The serosa was intact and the mesentery was uninvolved.

**Figure 3:**
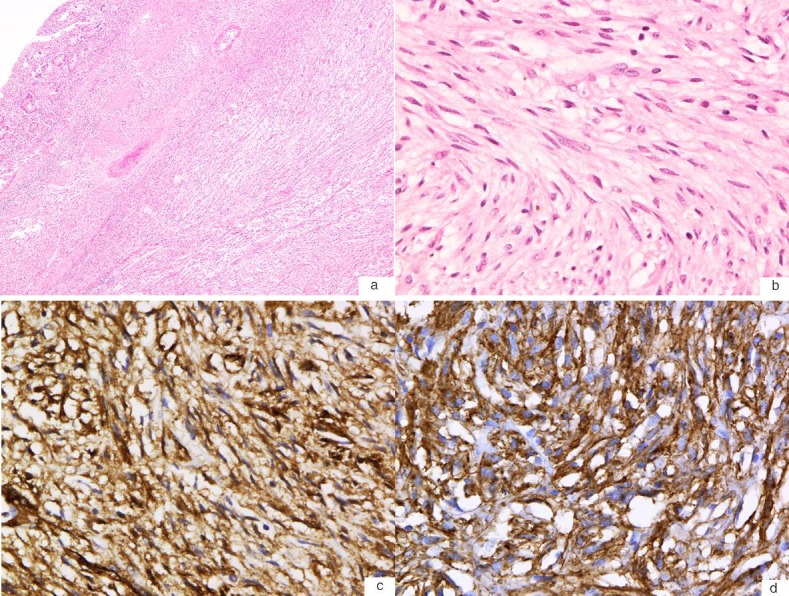
(a) Hematoxylin and eosin stain of the GIST shows a spindle cell neoplasm with a relatively low cellular density (40X). (b) High-power magnification (400X) shows that the neoplasm is comprised of interlacing bundles of bland spindle cells with fibrillary eosinophilic cytoplasm with a paucity of mitotic figures (0 mitosis per high-power field). (c) Immunohistochemistry stain shows that the tumour cells are immunoreactive for CD34 (400X). (d) Immunohistochemistry stain also shows that the tumour cells are immunoreactive for c-kit (CD117) (400X).

Within an hour after the colonoscopy, he developed severe left upper quadrant abdominal pain with distension and vomiting. Abdominal x-ray did not reveal free air. CT abdomen with IV and oral contrast showed a large left upper quadrant jejunal intussusception extending over a length of 25 - 30 cm, containing mesenteric fat and vessels, with associated gastric distension (Fig. 1a - e). At emergency laparotomy, 45 cm of small bowel intussusception was resected en bloc. Intra-operative findings revealed a dilated loop of small bowel that contained an intussuscepted portion of small bowel, the walls of which appeared dusky with no evidence of perforation. Gross examination of the resected specimen confirmed intussusception of small bowel with a 2.5 cm tumor within the submucosa and muscularis propria (Fig. 2a, b). Microscopically the tumor consisted of a proliferation of bland spindled cells arranged as interlacing fascicles (Fig. 3a, b). The mitotic count was 0 per high power fields (hpf) and necrosis was absent. The attached mesenteric lymph nodes were negative for metastasis and the margins were free of tumor. By immunohistochemistry, the neoplastic cells were positive for vimentin, CD34 + (Fig. 3c) and c-kit (CD117) (Fig. 3d), while negative for desmin, anti-smooth muscle actin and S-100. The Ki-67 proliferation rate was less than 3%. The features were characteristic of a gastrointestinal stromal tumor (GIST), with a low risk of progressive disease predicted on the basis of tumor site, tumor size and mitotic count [4]. He was discharged home 6 days after his surgery. No further specific treatment was recommended for his GIST but surveillance was recommended clinically and with abdominal imaging. A small bowel obstruction at the site of his anastomosis one month after surgery resolved with bowel rest alone. Six months after his surgery, he has not had any further GI bleeding while on dalteparin.

## Discussion

This case illustrates the complex presentation of a patient with a jejunal GIST presenting with obscure, overt gastrointestinal (GI) bleeding and an acute abdomen secondary to jejunal intussusception shortly after a colonoscopy. Complicating his presentation were colonic angiodysplasias, which were another source of GI bleeding, and venous thromboses requiring anticoagulation, which may have predisposed him to bleeding.

Obscure GI bleeding may account for approximately 5% of all GI bleeding, and is frequently due to a lesion in the small bowel. Angiodysplasia is the most frequent cause of mid-gut bleeding in approximately 70% of cases. However, as in our patient, the presence of angiodysplasias does not preclude another source of GI bleeding. Small bowel tumours may be found in 10-20% of cases of obscure GI bleeding [5]. The relative frequency of the etiology of small intestinal bleeding has not been well-defined in large population-based studies, but is age-dependent. Those younger than 40 years are more likely to have small intestinal tumors (lymphomas, carcinoid tumours, adenocarcinoma, polyps from hereditary polyposis syndrome), Meckel’s diverticulum, Dieulafoy’s lesion and inflammatory bowel disease, while those older than 40 years are more likely to have bleeding from vascular lesions (up to 40% of all causes), and non-steroidal anti-inflammatory drug-induced small bowel disease [6]. In the clinical setting of obscure GI bleeding, the diagnostic value of capsule endoscopy and double balloon enteroscopy has been shown to be similar and complementary [7-9]. For instance, a meta-analysis of 11 studies involving 757 subjects with obscure GI bleeding found that the diagnostic yield for capsule endoscopy and double balloon enteroscopy was 60% and 57%, respectively, for small intestinal lesions [7].

GISTs are the most common mesenchymal neoplasm of the GI tract, but are the least common of small intestinal malignant neoplasms, with an annual incidence of 1.2 cases per million population [10]. In a population-based study of the incidence of malignant small bowel tumours, carcinoid tumours and adenocarcinomas were the most common histological subtypes, followed by lymphomas and sarcomas [11]. Most GISTs are composed of spindle cells, though 20-30% are mainly composed of epitheloid cells [12]. C-kit (CD117) expression is a defining feature of GISTs, as immunohistochemical staining for this tyrosine kinase receptor is positive in 95% of GISTs [13]. C-kit is activated by the binding of the stem cell factor, KIT ligand, and 75-80% of GISTs have KIT mutations, while 8% have platelet-derived growth factor receptor a polypeptide gene (PDGFRA) mutations [14, 15]. Mutations in these genes result in functional protein changes, which result in constitutional activation [12].

The predominant site for GISTs is the stomach (60-70%), followed by the small intestine (25-35%), and less than 5% occur in the rectum, esophagus, omentum and mesentery [13, 16]. Within the small intestine, 17.7% are in the duodenum, 47.6% in the jejunum, and 34.7% in the ileum [10]. In two large series, approximately 40% of patients with GISTs presented with pain, 38% with an abdominal mass, and in a further 30% GI bleeding was the initial symptom [17, 18]. GISTs can cause intussusceptions or rarely intestinal obstruction, both of which are uncommon presentations of GISTs because of their tendency to grow in an extraluminal fashion [2].

In our patient, radiologic imaging and endoscopic visualization of the small bowel failed to locate the source of his GI bleeding. This is likely because endoscopically, GISTs appear as submucosal lesions, with an oval or smooth shape, and with normal overlying mucosa with occasional central ulceration and a firm consistency on compression [2, 12]. Although double balloon enteroscopy allows for biopsies, a histological diagnosis of GIST can be missed on routine mucosal biopsy because of its submucosal origin, and biopsy sampling may result in massive bleeding because of its hypervascularity [1, 19]. Pre-operative biopsies of GISTs have been found to be diagnostic in only 50% of cases [18, 20]. In this patient, it is likely that the region of jejunal granularity seen on double balloon enteroscopy formed part of the GIST, and the submucosal localization of this GIST made endoscopic diagnosis difficult.

While endoscopic procedures and small bowel imaging had failed to diagnose this patient’s jejunal GIST, it was the development of a surgical emergency that led to the diagnosis. Only three cases of adult colo-colonic intussusception following colonoscopy have been reported [21-23]. However, there have been no reported cases of small bowel intussusception presenting after colonoscopy. A proposed mechanism for the pathogenesis of idiopathic (or iatrogenic) intussusception in adults post-colonoscopy is hyperperistalsis, which leads to venting of gas and emptying of the insufflated colon after colonoscopy [21]. Another possibility is that intussusception may be induced by post-polypectomy mucosal edema acting as a lead point [22]. In our patient, the jejunal intussusception was in the context of a GIST, which altered normal bowel peristalsis, and its temporal relationship after the colonoscopy may have been coincidental or may be related to the inflation of the small bowel from ileal intubation and manipulation, though this has not been previously reported.

Surgical resection is recommended in almost all cases of adult intussusception, because of the high prevalence of structural lesions and the significant risk of underlying malignancy. However, optimal treatment with either reduction or primary resection remains controversial. Weilbacher et al [24] proposed the concept of mandatory primary resection without reduction, because of the high incidence of underlying malignancy and the theoretical risk of intraluminal seeding from reduction. However, resection requires the excision of a long segment of small bowel, which can compromise mesenteric vessels. Baig et al proposed that gentle intraoperative reduction can be attempted before resection to minimize unnecessary excision of healthy bowel [25]. In our patient, he had 45 cm of small bowel resected. Reduction was not initially attempted given the questionable viability of his bowel wall and because of concern of an underlying structural lesion causing his recurrent GI bleeding and acting as the apex of his small bowel intussusception.

GISTs in the small intestine are more aggressive than those in the stomach, with malignant behavior detected in 40-50% of small bowel GISTs compared with 20-25% of gastric GISTs [13]. Complete en bloc resection is the primary treatment for localized GISTs, with the goal for complete tumor removal with clear resection margins. Following complete resection, CT surveillance every 3 - 6 months for 3 - 5 years, and annually thereafter is recommended by the National Comprehensive Cancer Network (NCCN) guidelines [26]. The NCCN endorsed the risk classification system proposed by Miettinen and Lasota, which is based on long-term follow-up data from over 1900 GIST patients from the pre-imatinib era, and which stratifies risk according to tumor size, number of mitoses per 50 hpf and tumor location [27]. A TNM (tumor-node-metastasis) staging system for GIST was recently developed by the American Joint Committee on Cancer and International Union Against Cancer [28]. Though not included in the TNM staging system, additional variables associated with recurrence include intraoperative findings of tumor rupture, invasion or peritoneal dissemination, which are “clinically malignant factors” that can identify patients who may benefit from adjuvant imatinib, a tyrosine kinase inhibitor, which has revolutionized the treatment of GIST [29]. Data from the recent SSGXVIII trial showed that in 400 patients with high-risk resected GIST (tumor size > 10 cm, mitotic count > 10/50 hpf, tumor size > 5 cm with mitotic rate > 5/hpf, or tumor rupture) at a median follow-up of 54 months, imatinib (400 mg daily) was associated with significant improvement in recurrence-free survival and overall survival [30]. In our patient, his GIST had no high risk features, and as such, surveillance with serial abdominal imaging was recommended.

In conclusion, this case presents an unusual case of a jejunal GIST, which presented with obscure, overt GI bleeding and was diagnosed only at laparotomy following jejunal intussusception post-colonoscopy. This case highlights the diagnostic difficulty and unusual presentation of a small intestinal GIST.
